# Brucellosis relapse causing thoracic aortic ulcers and aneurysm formation: a case report

**DOI:** 10.1186/s12879-021-07005-7

**Published:** 2022-01-10

**Authors:** Shuai Li, Qiang Wang

**Affiliations:** Department of Infectious Disease, Shanxi Provincial People’s Hospital, Taiyuan, China

**Keywords:** Brucellosis, Relapse, Thoracic aortic ulcer, Aneurysm

## Abstract

**Background:**

Brucellosis is an infectious disease caused by *Brucella* spp, which can involve the cardiovascular, digestive, and respiratory systems. Cardiovascular involvement is a rare occurrence, it has an extremely high mortality rate.

**Case presentation:**

A 67-year-old Chinese man presented with thoracic aortic multiple ulcers and partial aneurysm formation that caused symptoms of left waist and left buttock pain. The man was admitted to our hospital due to abdominal aorta pseudoaneurysms 5 years ago. The diagnosis was made by thoracic computed tomography angiography (CTA), previous history, and positive culture of *Brucella*, and the patient was successfully treated by thoracic aortic covered stent-graft implantation and specific medical treatment.

**Conclusions:**

People who have a history of contact with cattle and sheep, should beware of the possibility of *Brucella* infection. If chest and abdominal pain occur, timely medical treatment is recommended, aortic aneurysm, the disease with a high risk of death, can be identified or excluded by CTA. Early treatment and prevention of disease progression are more beneficial to patients.

## Background

*Brucella* belongs to Gram-negative bacteria, often parasitizes in sheep, cattle, and other animals. In China, it is most common that infecting and causing disease through sheep. Brucellosis is a common zoonosis that is prevalent worldwide and can affect various systems throughout the body. We reported a case of previously treated brucellosis combined with abdominal aortic pseudoaneurysms. He was present thoracic aortic multiple ulcers and partial aneurysm formation due to brucellosis relapse. We performed thoracic aortic stent implantation under general anesthesia. Such cases have not been reported.

## Case presentation

A 67-year-old Chinese man, with long-standing high blood pressure and type 2 diabetes mellitus, presented with left waist and left buttock pain for 1 week. Blood cultures grew *Brucella*, and the CTA revealed multiple thoracic aortic ulcers and partial aneurysm formation. He was diagnosed with brucellosis. Five years ago, he suffered from an abdominal aorta pseudoaneurysm, and underwent surgery. During hospitalization, blood culture grew *Brucella*. After departure from the hospital, he was treated with a combination of rifampicin and doxycycline, but blood cultures grew *Brucella* many times. The patient described that he worked in a restaurant in Inner Mongolia five years ago, but he has no contact history with cattle or sheep since he was discharged from hospital. After this admission, the patient’s body temperature, blood pressure, heart rate and respiration were normal, and no other abnormalities were found in physical examination. Laboratory studies revealed the white blood cell count (WBC) was 5.15 × 10^9^/L, the hemoglobin(Hb) was 122 g/L, the platelet count (PLT) was 152 × 10^9^/L, the C-reactive protein (CRP) was 17.28 mg/L, the erythrocyte sedimentation rate (ESR) was 20mm/h, the triglyceride was 2.33 mol/L, the low-density lipoprotein was 3.42 mol/L, normal electrolyte and coagulation indexes, normal electrocardiogram (ECG). The blood creatinine was 125.02 µmol/L. The renal artery imaging showed that the blood perfusion of both kidneys was fair, the uptake and removal of imaging agents in the cortical cortex of both kidneys were reduced, the function was mild to moderate impaired, and the upper urinary drainage of both kidneys was fair. The glomerular filtration rate was 54.1 mL/min (normal > 73.0 mL/min), 30.7 ml /min on the left side and 23.4 mL/min on the right side. Explain to the patient and family, extensive use of contrast agents may worsen kidney injury during the operation, but the patient may at any time for thoracic aortic ulcer aggravating life-threatening, or intimal plaque off cause distal arterial embolism, the patient and family said they understood the disease and the risks and agreed to the operation, we performed thoracic aortic stent implantation under general anesthesia. Seen through imaging by catheter angiography: the diameter of the distal aortic arch of the left common carotid artery was about 2.9 cm, the nearest ulcer was located on the opposite side of the left subclavian artery and the minor curvature of the aortic arch, the farthest thoracic aortic ulcer was located in the thoracic 9–10 intervertebral space. A 32–160 thoracic aortic stent (ANKURA, XJZDZ32160) was placed along the guide-wire at the level of the ascending aorta. The beginning of the coated end was located at the distal opening of the left common carotid artery. A 32-26-200 aortic stent (ANKURA, TAA3226B200) was placed along the guide-wire, and the distal position of the stent was about 2.5 cm beyond the ulcer at the lowest end of the thoracic aorta. A 9–39 mm stent (Micro Medical, ICLC9039L) was placed along the guide-wire with the proximal end beyond the coated skin frame of the thoracic aorta and the distal end within the left subclavian artery. After then, catheter angiography showed: ascending aorta, aortic arch, truncus brachiocephalicus, left common carotid artery, left subclavian artery blood flow were smooth, and the blood vessel wall is smooth. The operation was successful. The patient’s vital signs remained stable throughout the operation. The patients were followed up 6 weeks after the surgery and had no complaints of any special discomfort.

## Discussion and conclusions

Brucellosis is a zoonotic infectious disease caused by *Brucella* spp. It is often caused by the ingestion of be contaminated undercooked meat and dairy products, and the contact history of related animals such as sheep and cattle [1], so the risk of the disease is higher in related occupations such as keepers and dairy workers. *Brucella* can cause multisystem involvement, but cardiovascular involvement is less common. Endocarditis, which occurs in 1–2% of cases [2], has a high risk of death, accounting for more than 75% of the deaths of brucellosis [3, 4]. *Brucella* is an intracellular parasitic bacterium. Bacteria and toxins play a major role in the acute phase, while delayed allergic reaction and the formation of granuloma are the main ones in the chronic phase [5]. Although *Brucella* can lead to endovasculitis if it affects blood vessels [5], the vascular complications of brucellosis are rare. In a review of 25 cases of brucellosis with aortic involvement, Kakkos et al. found that 65% of the abdominal aorta and 23% of ascending aorta were involved [6]. Only a small number of cases have been reported in the past 5 years [7–12], and only one case has been reported recurrent in the past 10 years, presenting as prosthetic valve endocarditis and aortic root infective pseudoaneurysms[13]. The aortic involvement typically showing the formation of major aneurysm or large aneurysm. Brucellosis causes severe inflammatory reaction by infection of abnormal vascular tissues, such as intimal defect caused by atherosclerosis [14], activating endothelial cells, up-regulating adhesion molecules, and secreting pro-inflammatory chemokines [15], leading to severe inflammatory reactions. As a result of *Brucella* infection, artery endothelium is damaged, and the middle layer is exposed to the blood flow, then forming arterial ulcers, aortic dissection, aortic aneurysm and aortic rupture.

At admission, the patient had left waist and left buttock pain for 1 week. Review with five years ago, the patient had visited our hospital due to *Brucella* abdominal aortic pseudoaneurysm. The symptoms were left upper abdominal pain for 1 month, transferred to the back pain for 20 days. He had a contact history of cattle, but no contact history of cattle has been found since his departure from the hospital. Therefore, the patient had similar symptoms, CTA examination showed multiple ulcers of thoracic aorta and the formation of partial aneurysm, which was most likely caused by the relapse of previously acquired infection, but the symptoms may be atypical due to the short course of disease.

**Fig. 1:**
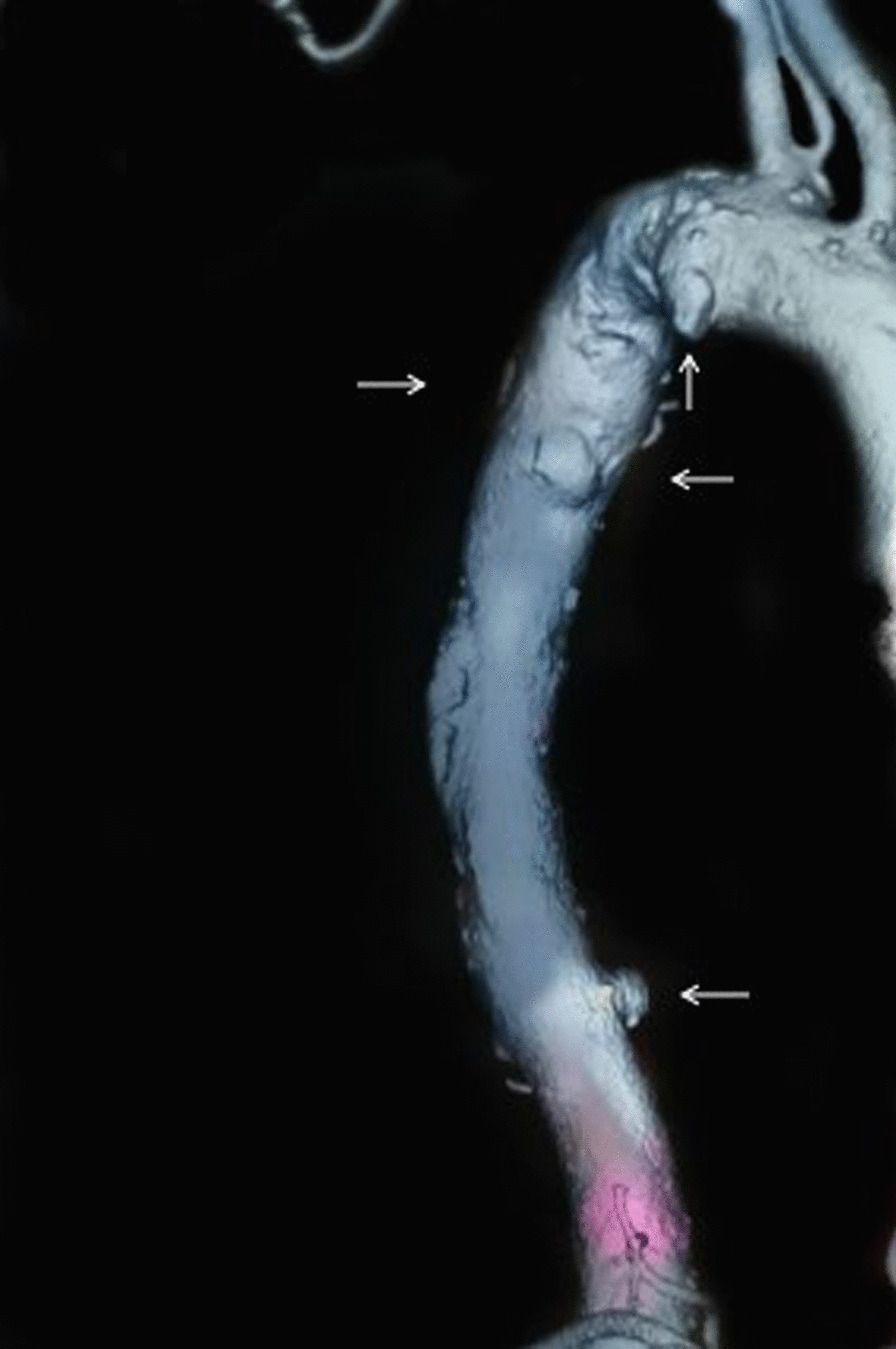
Computed tomography angiography (CTA): Multiple penetrating ulcers in thoracic aorta, partial aneurysm formation

In conclusion, as a case of multiple thoracic aortic ulcers and partial aneurysm formation due to brucellosis relapse, and the patient was successfully treated by thoracic aortic covered stent-graft implantation and specific medical treatment. However, this case still has some limitations: (1) Intraoperative pathological analysis was not obtained, and the diagnosis was based on CTA, previous history and blood culture results; (2) The postoperative follow-up was only 6 weeks, we will further follow-up. However, people who have a history of contact with cattle and sheep, should beware of the possibility of Brucella infection. Rose bengal plate test (RBPT) is feasible to conduct preliminary screening. During treatment follow-up, non-specific tests such as ESR and CRP should also be used besides for serological tests [16]. If chest and abdominal pain occur, timely medical treatment is recommended, aortic aneurysm, the disease with a high risk of death, can be identified or excluded by CTA. Early treatment and prevention of disease progression are more beneficial to patients (Fig. 1).

## Data Availability

The datasets used during the current study are available from the first author upon reasonable request.

## Abbreviations

CTA: Computed tomography angiography
WBC: White blood cell count
Hb: Hemoglobin
PLT: Platelet
CRP: C-reactive protein
ESR: Erythrocyte sedimentation rate
ECG: Electrocardiogram
RBPT: Rose Bengal plate test
